# Is AMOEBA a Good Force Field for Molecular Dynamics Simulations of Carbohydrates?

**DOI:** 10.1021/acs.jcim.5c00442

**Published:** 2025-05-20

**Authors:** Mawuli Deegbey, Ethan W. Sumner, Valerie Vaissier Welborn

**Affiliations:** † Department of Chemistry, 1757 Virginia Tech, Blacksburg, Virginia 24061, United States; ‡ Macromolecules Innovation Institute, Virginia Tech, Blacksburg, Virginia 24061, United States

## Abstract

Over the years, molecular dynamics (MD) simulations have been employed in the study of carbohydrates, with force fields such as CHARMM, AMBER/GLYCAM, and GROMOS. Although these force fields have achieved considerable success and played a pivotal role in our understanding of carbohydrate chemistry, growing interest has emerged in incorporating polarization effects to enhance the accuracy of simulations. In this perspective, we contemplate the advances that have been made in nonpolarizable and polarizable force fields to extract the key factors controlling accuracy in MD of carbohydrates. We find that the extreme hydrophilicity and conformational flexibility of carbohydrates pose challenges for most force fields. Overall, a force field suited for carbohydrates needs to include a water model developed consistently with the solute parameter sets, a soft van der Waals repulsion term at short distances, and polarization (whether implicit or explicit). We find that AMOEBA improves the prediction of hydration shell structure and dynamics, hydrogen bonding, and kinetics of diffusion, although it remains largely untested for conformational flexibility and glycosidic linkages. Nevertheless, AMOEBA’s recent success in modeling monosaccharides without revisions of the potential energy functions or water model presents a promising avenue for future research. Such advances will provide deeper insights into the structure, dynamics, and interactions of these biologically and industrially relevant macromolecules.

## Graphical abstract

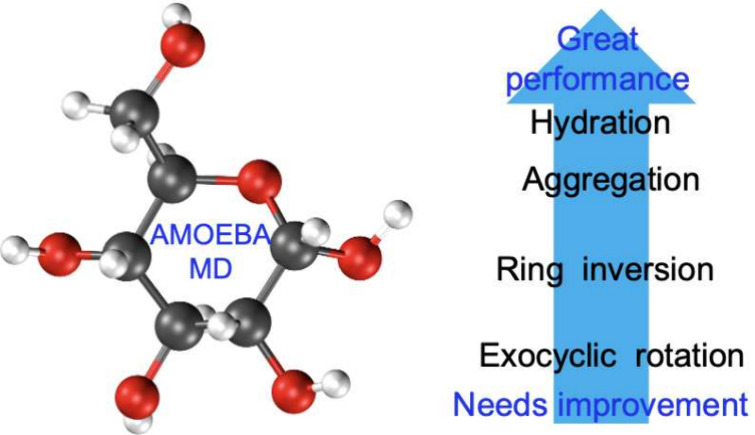

## Introduction

Carbohydrates are biomolecules that contribute to cell structure, signaling pathways, disease progression, and energy storage in living organisms.^1–4^ The most fundamental forms of carbohydrates are mono- and disaccharides that contain multiple hydroxyl groups. These hydroxyl groups make carbohydrates highly polar with a strong affinity for water. Further, the position and orientation of the hydroxyl groups define steroisomers with different physicochemical properties. For example, α-d-glucose exhibits properties different from β-d-glucose due to the change in orientation of the hydroxyl group on the anomeric carbon (Figure 1a). Stereochemistry also affects the assembly of carbohydrate into longer chains through the formation of glycosidic linkages. Indeed, α-d-glucose will form a helical polysaccharide -amylose- through (1 → 4) linkages, while β-d-glucose will form a linear polysaccharide -cellulose- through (1 → 4) linkages (Figure 1b).

**Figure 1:**
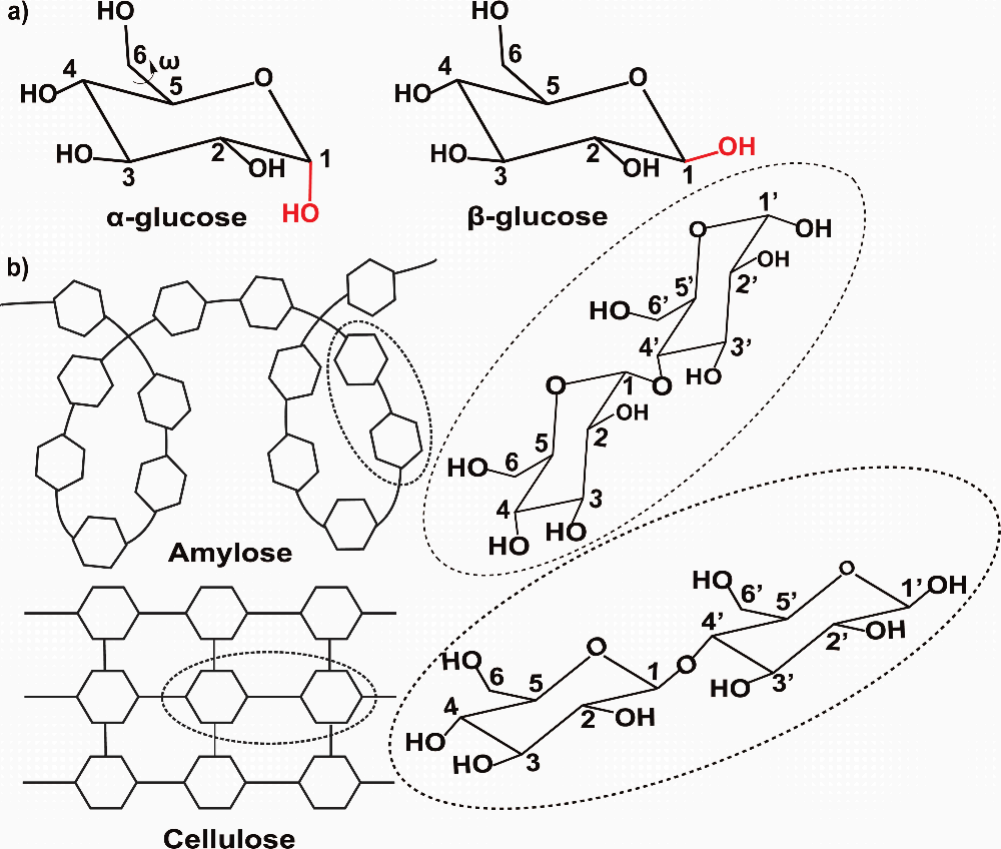
a) Structures of α- and β-d-glucose, highlighting in red the axial and equatorial positions of the hydroxyl group on C_1_. b) Structures of amylose and cellulose, rising from the α-1,4 and β-1,4 linkages of glucose, shown on the side.

Carbohydrates are also reactive.^5,6^ Common derivatives found in living organisms include amino, acidic, deoxy carbohydrates, and carbohydrate phosphates.^7–10^ These natural modifications have inspired many to synthesize novel, functionalized, carbohydrates to better control their interactions with proteins, lipids, and other biomolecules and to create new building blocks for (bio)­material design.^11–14^

The modularity of carbohydrates makes them relevant across research fields, and a strong structure-to-function relationship is necessary to optimize the synthesis, characterization, and design of carbohydrate-based systems. Atomistic modeling is a core component of this effort as it ties molecular properties to the observed behavior of the resulting macromolecules or materials.^15^ In particular, classical Molecular Dynamics (MD) is a great tool to capture carbohydrates’ inherent flexibility and their affinity for water.^16^ However, the accuracy of classical MD simulations depends on the force field used to compute the potential energy and the corresponding forces. The ideal force field can be defined as one that incorporates the minimal number of potential terms to be accurate but efficient. In principle, if the right physics is taken into account, the functions in this ideal force field can readily be transferred to any arbitrary molecule. In practice, force fields consist of empirical functions that have been developed to model interactions in proteins, and the transfer to carbohydrates is not trivial.^17–19^ However, our fundamental knowledge of molecular carbohydrate chemistry has improved by attempts to use and adapt existing force fields. This exercise enabled the identification of key factors to include such as polarization, torsional flexibility, and hydrogen bonding. In this context, the more recent polarizable force fields^20,21^ seem better suited to model carbohydrate chemistry.^22,23^ Indeed, while polarization has been added to improve the accuracy of MD for proteins, it is a fundamental component of modeling any molecule in water including carbohydrates. In other words, polarizable force fields are a step closer to becoming the ideal force field, describing enough physics to be generalizable. In particular, the Atomic Multipole Optimized Energetics for Biomolecular Applications (AMOEBA) force field incorporates physics-based functions that were proven more directly transferable to carbohydrates.^21,23,24^ AMOEBA differs from other force fields in three major terms: (i) polarization, or induced electrostatics, with the inclusion of an induced dipole scheme, (ii) permanent electrostatics with the incorporation of multipoles at atomic positions instead of single point charges, and (iii) van der Waals interactions with the adoption of the buffered 14–7 Halgren potential that yields softer repulsion at short distances compared to the traditional 12–6 Lennard-Jones potential.^25^

In this Perspective, we review key carbohydrate properties that led to the identification of limitations in existing force fields and further drove the development of polarizable force fields. We highlight the performance of the AMOEBA force field, as it appears to be better able to tackle highly hydrophilic carbohydrates, despite its only recent use in the field.

## Predicting Carbohydrates’ Hydration Shell Structure

The numerous hydroxyl groups in carbohydrates facilitate bonding with water molecules through strong hydrogen bonds and electrostatic interactions.^26,27^ These molecules are tightly bound to the carbohydrate and help shape its chemical and structural properties.^28,29^ As a result, force fields need to correctly balance carbohydrate-water interactions with carbohydrate-carbohydrate and water–water interactions to accurately predict their collective behavior.^17,30^ In MD, there are two quantities that are often computed to characterize the structure of water around a given solute, thereby serving as a benchmark across studies: radial distribution functions (RDFs)^31^ and the orientation order parameter, *q*.^32,33^ RDFs are average distributions of particles around a central particle. In this case, it characterizes how the packing of water molecules varies as a function of the distance from the carbohydrate. *q* is defined as

1 $$q=1-\frac{3}{8}\overset{n}{\underset{j>k}{\sum }}{\left(\cos\left(\psi_{ijk}\right)+\frac{1}{3}\right)}^{2}$$

where ψ_*ijk*_ is the angle formed between the oxygens of neighboring water molecules *i*, *j*, and *k*. *q* measures the propensity of water to adopt a tetrahedral arrangement, a hallmark of hydrogen-bond networks in liquid water. Branca et al. has shown experimentally that trehalose, sucrose, and malose have a destructuring effect on the tetrahedral hydrogen-bond network of water.^34,35^ MD needs to reproduce this behavior for accurate predictions of carbohydrate properties in water.^36^

### Early Force Field Applications to Carbohydrates

The Chemistry at Harvard Macromolecular Mechanics (CHARMM)^37^ force field was used in the late eighties, in combination with the Simple Point Charge (SPC) water model, to investigate glucose hydration.^27^ The authors computed glucose hydroxyl-water oxygen RDFs and found that they were qualitatively similar to oxygen–oxygen RDFs in bulk water. Specifically, they found sharp and narrow first hydration shell peaks at 2.70 Å for O_2_, O_3_, and O_6_, and at 2.75 Å for O_1_ and O_4_. These well-defined peaks were characterized by deep minima, suggesting that the water molecules near the hydroxyl groups of glucose were highly localized. Despite variations in the position of the first minimum, the peak integral was comparable across hydroxyl groups. The corresponding coordination numbers (CNs) ranged from 3.0 to 3.6, with O_2_ and O_3_ being the most coordinated. The same authors carried out a similar study on disaccharides, maltose and xylose, using CHARMM and the better developed TIP3P water model.^38^ A greater number of hydrogen bonds with water was observed in maltose with less structuring around the solute. In the early 2000s, Lerbret et al. also investigated the hydration properties of maltose and sucrose, as well as those of trehalose.^39^ They used a revised version of the CHARMM force field^40^ with the Extended Single Point Charge (SPC/E) water model. Based on their analysis of RDFs and CN, the authors found that trehalose binds more water molecules than the other disaccharides, significantly impacting the local water structure. Although this result is consistent with the superior cryoprotective properties of trehalose,^41–43^ Brady et al. pointed out that the choice of the water model plays a significant role in determining the extent of hydrogen bonding in MD.^38^ A recent benchmark study on water models in protein–glycosaminoglycan systems confirms the influence of water model selection on the extent of hydrogen bonding.^44^ In this case, the TIP4P model displayed a significant increase in hydrogen bond formation compared to that of other water models over 10 μs MD simulations.

More importantly, the water model, often developed independently, needs to cooperate with the solute parameter sets. The importance of the match between water model and force field is exemplified in another early MD study of α-glucose.^45^ In this case, Glennon et al. used the Assisted Model Building with Energy Refinement (AMBER) force field^46,47^ with the SPC/E water model. The AMBER simulations revealed that the position of the first peak for each glucose oxygen ranged from 2.68 Å to 2.73 Å, similar to the predictions from the CHARMM force field with the same water model. AMBER also predicted O_2_ and O_3_ to be the most coordinated, but the CN was much lower. This difference was associated with variations in the flexibility of the hydroxyl groups between the two force fields, where AMBER allows for greater variability in orientation. However, the AMBER simulations also showed a second solvation shell around 5.0 Å of O_5_, a feature absent in previous CHARMM simulations with the TIP3P water model. This is consistent with an earlier study that found TIP3P to result in a less structured carbohydrate hydration shell compared to SPC.^38^ The missing hydrogen bonds in TIP3P simulations translated in erroneous diffusion coefficients.^38^ After careful analysis, they concluded that the partial charges of the solute need to be balanced with those of the water model to adequately capture the relative strength of water-solute and water–water interactions.^38^ Although not as problematic for MD simulations of proteins, the mismatch between the empirical functions of existing force fields and independent water models may result in erroneous solution properties for carbohydrates. That is because carbohydrate-water and water–water interactions are of a similar strength, which requires accurate balancing at short-range. In summary, the extreme hydrophilicity of carbohydrates made use of the existing empirical force field difficult, and carbohydrate-specific developments were necessary.

GLYCAM is one such set of parameters, developed specifically for carbohydrates using the empirical potential energy functions of the AMBER force field.^48^ The authors derived GLYCAM within the AMBER framework to take advantage of the asymmetric torsional potentials that are likely to better capture the flexibility of glycosidic linkages in addition to the flexibility of the hydroxyl groups described above. Using the GLYCAM 2000a force field^49^ with the TIP3P water model, Corzana et al. computed RDFs of α-d-isomaltoside.^50^ They report a first hydration shell at approximately 3.0 Å with a poorly defined secondary hydration shell. The authors noted that such distance was greater than the average separation observed in carbohydrate crystal structures^51^ (2.77 Å).

However, Kirschner et al. developed a revised version of GLYCAM, called GLYCAM06, and observed significant improvements in the RDFs when used with TIP3P.^52^ Indeed, they reported a first hydration shell at 2.75 Å, closely matching experimental data, and a well-defined secondary hydration layer at approximately 5.0–5.5 Å. The authors attributed these refinements in GLYCAM06 to the incorporation of AMBER-consistent van der Waals parameters for hydroxyl oxygen atoms, leading to more accurate solute–solvent interactions.

### Polarizable Force Fields

Over the years, the CHARMM, AMBER, GLYCAM, and GROMOS force fields have been revised, and the accuracy of MD simulations, including simulations of carbohydrates, correspondingly improved.^16,29,53^ As discussed above, a majority of the reported improvements for carbohydrates came from employing a water model that synergizes with the force field and the tuning of van der Waals parameters and torsion terms. However, newer studies showed that these additive force fields cannot fully describe solvent and hydrogen bonding effects without the inclusion of polarization.^15,22,53,54^

In 2016, Pandey and Mallajosyula specifically investigated the effect of polarization on the solvation dynamics of six monosaccharides.^22^ The authors compared the additive CHARMM force field with the additive water models TIP3P, TIP4P, and TIP5P^55–57^ to the polarizable Drude force field and the polarizable SWM4 water model.^58^ The water-monosaccharide RDFs revealed a sharp peak at 2.80 Å, indicating that the water molecules are highly localized within the first hydration shell. Among the models analyzed, the polarizable SWM4 water model demonstrated an extended coordination sphere and a higher CN, reflecting greater-range interactions compared with the additive models. The probability distribution of the tetrahedral parameter, *P*(*q*), within the first hydration shell provided further insight into the structural organization of water. For pure water, *P*(*q*) exhibits a bimodal distribution with a strong peak at 0.8 (ordered tetrahedral) and a smaller peak at 0.5 (disordered tetrahedral). In the presence of monosaccharides, the low-q peak (0.5) increased in amplitude, while the high-q peak (0.8) decreased, indicating a disruption in the local tetrahedral arrangement. This effect was most pronounced with TIP5P and SWM4, due to their superior hydrogen-bonding capabilities and higher water-bridge occupancies within the first hydration shell. Although *q* is a local descriptor that pertains to atomistic simulations,^33^ the range of disruption from monosaccharides (∼3.5 Å) aligns with terahertz (THz) spectroscopy data (3.0–4.0 Å).^59^ In contrast, TIP3P failed to reproduce the bimodal distribution, which exposes its limitations in accurately representing carbohydrate hydration.

In a recent study by our group, we tested the AMOEBA polarizable force field to investigate the solution properties of similar monosaccharides.^23^ AMOEBA has already demonstrated high accuracy in modeling water and protein dynamics, in large part because it includes its own water model, developed consistently with the other parameter sets.^21^ In this study, we presented new parameters for monosaccharides, computed using the Poltype 2 code,^60^ without further modifications. This is important, as it shows the transferability of AMOEBA to systems for which it was not initially developed. The RDFs revealed first peaks at 2.85 Å, sharper and narrower than those computed with the Drude force field. This yields a more ordered and localized hydration shell. For reference, the same simulations were run with the AMOEBA force field, turning off the mutual polarization scheme, and the RDFs exhibited broader and shorter peaks, indicative of a less-defined hydration shell. Therefore, it is likely that the difference between the Drude and AMOEBA-computed RDFs is due to the different treatment of polarization effects. Interestingly, we also observed a denser hydration shell with AMOEBA, a feature not observed in previously reports.^22^ The increased density was attributed to softer van der Waals repulsion at short distances, enabled by the buffered 14–7 Halgren potential in AMOEBA, which cooperates well with the water model for an inherently consistent and transferable model. While we can rationalize the source of differences between force fields in predicting RDFs, it is difficult to validate these data against experimental data. In the next section, we review the ability of the aforementioned force fields to predict diffusion kinetics in water, which helps to decide the force field accuracy.

## Diffusion Kinetics in Water

In the previous section, we discussed the accuracy of various force fields in predicting the hydration shell structure of carbohydrates. This is a fundamental stepping stone to predicting carbohydrates’ reactivity and mobility. Therefore, recent studies often provide kinetic benchmarks for further force field validation. In this context, Sauter and Grafmüller modeled glucose at different concentrations (0.25, 0.5, 1, 2, 3, and 4 mol.kg^–1^) with the GROMOS 56a6_CARBO_, CHARMM, and GLYCAM force fields.^54^ The authors reported aggregation of glucose within nanoseconds in all cases, which is nonphysical because all simulations were below the solubility limit. This was confirmed by Batista et al., who employed the GROMOS 56a_CARBO_ force field for similar experiments.^61^ Sauter and Grafmüller quantified the aggregation of glucose by computing the center-of-mass (COM) RDF. They saw that the CHARMM/TIP3P and GROMOS 56a6_CARBO_/SPC force fields exhibited minor variations with concentration, contrary to expectations. Although GLYCAM/TIP3P correctly showed significant changes in RDF with concentration, it yields to overaggregation, even at low concentrations—an effect not observed in the other force fields. This outcome was attributed to the large Lennard-Jones parameters in GLYCAM (ϵ = 0.457730 for all C) compared to CHARMM (ϵ = 0.133888 for all C and 0.234304 for C_6_). Overall, the combination of the GLYCAM force field with the TIP5P water model performed better, although aggregation was still observed.

Sauter and Grafmüller also computed diffusion coefficients, *D*, which were overestimated with CHARMM/TIP3P, GLYCAM/TIP3P, and GROMOS56a6_CARBO_/SPC. Batista et al. also reported an overestimation of *D* at higher concentrations with GROMOS 56a_CARBO_.^61^ Similarly, simulations using the CHARMM C35 force field demonstrated a significant overestimation of diffusion coefficients for glucose and trehalose compared to experimental data.^62,63^ As for aggregation, improved agreement was observed with GLYCAM/TIP5P, although *D* was now underestimated. Since TIP5P includes implicit polarization effects, this suggests that polarization is key to accurate predictions of aggregation and diffusion kinetics. This was confirmed by Patel et al., who reported density predictions with an average error of 0.11% across varying concentrations and temperatures with the Drude force field.^64^ However, while *D* at lower concentrations aligned well with experimental data, higher concentration simulations continued to exhibit significant deviations, mirroring the challenges seen with additive force fields. Yang et al. further optimized the Drude force field by incorporating quantum mechanical data on glucose-water and glucose–glucose interactions.^65^ These refinements yielded notable improvements in the diffusion kinetics. However, despite these enhancements, the calculated *D* for both glucose and water remained overestimated at high glucose concentrations (3 M and 5M) compared to experiments, suggesting persistent inaccuracies in the solute–solute and solute–solvent interactions. The authors also observed different *D* for the SWM4 and SWM6 water models,^65^ reinforcing the need for an optimized water model consistent within each force field.

In our AMOEBA study,^23^ we did not observe aggregation of glucose at low or high concentrations, which readily improves predictions compared to the CHARMM, GROMOS, and GLYCAM force fields. We attributed this effect to the thin and dense hydration shell around the monosaccharides. Indeed, AMOEBA predicts a higher number of water molecules at a closer range compared to other force fields, which shields glucose from aggregation. Correspondingly, our calculated *D* agreed better with experiments, though slight underestimations were still observed.^23^ Nevertheless, AMOEBA stands out by outperforming previous additive and polarizable force fields in predicting kinetics properties.

## Force Field Validation beyond X-ray Data: The Emergence of Terahertz Spectroscopy

A big component of force field validation comes from the comparison between average computed geometries and crystallography, primarily X-ray, data. Although MD simulations of carbohydrates in water do not reproduce the operating conditions in crystallography, this approach has proven valuable to verify the geometry (i.e., bond length, angle, torsion, etc.) of the solute. Solution NMR enables further validation of exocyclic torsion flexibility,^66–69^ as we discuss in the next section. However, it is not trivial to validate the hydration shell structure and dynamics. Recently, we have observed the emergence of THz spectroscopy, which enables the capture of a broad range of molecular dynamics, from slow molecular tumbling to ultrafast hydrogen bond dynamics.^70,71^ For example, Havenith et al. used THz spectroscopy to investigate the influence of carbohydrates on the dynamics of water.^59^ They compared the effects of glucose, trehalose, and lactose on the subpicosecond dynamics of hydration shell waters. They found that carbohydrates increase the THz absorption coefficient of these waters by 2–4% compared with bulk water. The changes in THz absorption were correlated to the number of carbohydrate-water hydrogen bonds. Further, they found that the hydration shell extended up to 3.7 Å for glucose and 6.5 Å for disaccharides, consistent with MD simulations. Similarly, Shiraga et al. investigated the effects of glucose, fructose, sucrose, and trehalose on the hydrogen bond network in water using THz time domain - attenuated total reflection spectroscopy.^26^ They defined hydrated water as molecules with relaxation times significantly longer than those of bulk water. They demonstrated that glucose and trehalose had a stronger hydration number than fructose and sucrose, respectively, even though they share the same number of hydroxyl groups. For instance, the hydration number of glucose nears 20, which is much higher than previous reports but consistent with another dielectric spectroscopy study.^70^ Further, Shiraga et al. found that the fraction of non-hydrogen-bonded water molecules increased with the concentration of saccharide, suggesting that saccharides promote hydrogen-bond network disruption. However, no significant differences were observed between glucose and fructose or sucrose and trehalose in terms of non-hydrogen-bonded water, which contrasts with the work of others.^34,35,39^

In summary, THz spectroscopy has the potential to provide alternative experimental data that are valuable for the development of force fields for carbohydrates. However, more work is necessary to interpret the spectra and provide consistent benchmarks to the community. As of now, THz spectroscopy contributes to expand our knowledge of the structural and dynamical hydration properties of carbohydrates but has not yet reached a consensus in the field.

## Exocyclic Rotation in Carbohydrates

As carbohydrates are often found in cyclic form, their interaction with other molecules, such as water, proteins, lipids, etc., is governed by the orientation of their exocyclic groups. So far, we have mostly discussed the ability of various force fields to capture the orientation of hydroxyl groups in water. However, the orientation of hydroxymethyl (CH_2_– OH) groups is also a key determinant of carbohydrate conformers. In MD, we measure the exocyclic dihedral angle ω = C_4_C_5_C_6_O_6_ to define three conformers: (i) gauche trans (gt) when 0° < ω < 120° (ii) gauche–gauche (gg) when −120° < ω < 0°, and (iii) trans gauche (tg) when ω < −120° or ω > 120° (Figure 2). ω can also be used to compute J-coupling constants with Karplus’ equations, which can be directly compared to NMR data.^66–69,72^

**Figure 2:**
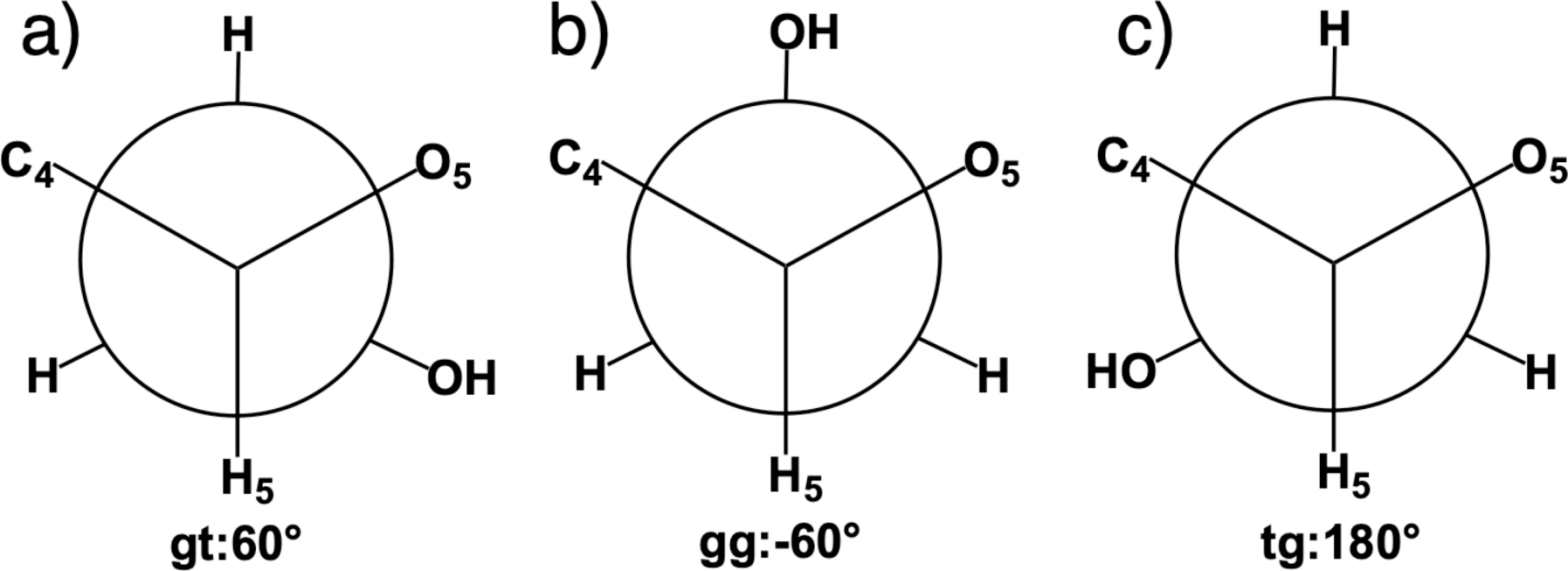
Three staggered hydroxymethyl rotational conformations. a) gt, b) gg, and c) tg.

Krauter et al. recorded the visited ω populations in MD simulations of β-d-glucose, β-d-mannose, β-d-galactose, and β-d-talose performed with the GROMOS 45A4/SPC force field.^73^ Their analysis revealed preferences for the gg and gt conformers in vacuum, with nearly zero population for the tg conformer. This was also the case in water for β-d-glucose (57% gg, 43% gt, and 0% tg) and β-d-mannose (52% gg, 48% gt, and 0% tg). For β-d-galactose and β-d-talose, all three conformers were populated in water, with a slight preference for the gg and gt conformers: 33% gg, 41% gt, and 26% tg and 39% gg, 39% gt, and 22% tg, respectively. Although the MD results for β-d-mannose in water were in good agreement with NMR data,^74–76^ the others did not match expectations. The rotational dynamics of ω were also estimated for each monosaccharide. The corresponding time constants for β-d-glucose (813 ps) and β-d-mannose (787 ps) were found to be significantly greater than those for β-d-galactose (175 ps) and β-d-talose (183 ps). This is qualitatively, but not quantitatively, consistent with experiments.^74–76^ Accordingly, the authors suggested further refinement of the torsional parameters in the GROMOS 45A force field.

In their GLYCAM06/TIP3P study, Kirschner et al. characterized ω in methyl α-d-glucopyranoside (α-d-GlcpOMe) and methyl α-d-galactopyranoside (α-d-GalpOMe).^52^ They found a 62%, 36%, and 2% occupancy for the gg, gt, and tg conformer of α-d-GlcpOMe, respectively, which closely aligned with the experimentally determined ratio of 53(57)%, 47(38)%, and 0(5) %.^75,77^ Similarly, the simulations for α-d-GlcpOMe (6% gg, 76% gt, and 18% tg) showed strong agreement with experiments (16(13)% gg, 75(70)% gt, and 9(17)% tg).^74,77^ These translated into the NMR coupling constants ^3^J_H5,H6R_ of 5.4 ± 1.7 Hz and ^3^J_H5,H6S_ of 2.9 ± 2.0 Hz for α-d-GlcpOMe and 7.9 ± 1.6 and 3.7 ± 1.8 Hz for α-d-GalpOMe, respectively. These values agree well with experimental data: 5.4(9) Hz and 2.3(9) Hz for α-d-GlcpOMe^74,77^ and are within the experimental range of 7.8–8.6 Hz and 3.7–6.0 Hz for α-d-GalpOMe.^74,75,77^ Therefore, while GLYCAM/TIP3P exhibited errors in capturing carbohydrate-water interactions, it performed well in terms of torsional flexibility of the hydroxymethyl groups.

Patel et al. investigated the effect of polarization on ω in d-glucose and d-galactose with the Drude force field.^64^ For d-glucose, they reported 52.3%, 30.5%, and 17.2% for the gt, gg, and tg of the α-anomer and 66.3%, 19.7%, and 14.0% for the β-anomer. This compares relatively well to the experimental ratio of 53.0%, 40.0%, and 7.0% for the α-anomer and 61.0%, 31.0%, and 8.0% for the β-anomer.^67,72^ For d-galactose, Drude predicted 56.0%, 20.0%, and 24.0% of the gt, gg, and tg conformers for the α-anomer and 61.2%, 15.5%, and 23.3% for the β-anomer. This deviates even more significantly from the experimental ratio of 74.0%, 3.0%, and 23.0% for the α-anomer and 72.0%, 3.0%, and 25.0% for the β-anomer.^67,72^ Despite overprediction of the gg conformer, Drude was reported more accurately than its additive counterpart, CHARMM. However, Chythra and Sairam reached the opposite conclusions, where the CHARMM additive force field outperformed Drude.^78^ Indeed, they computed ω distributions in α/β-d-glucose, α/β-d-galactose, α/β-l-idose, and β-d-manose and found that the additive CHARMM force field generally aligned well with experimentally derived NMR-J-coupling constants.^67,79–83^ In contrast, the polarizable Drude force field oversampled gt, deviating from known data, particularly for α/β-d-galactose and α/β-l-idose. For β-l-idose, the Drude force field also undersampled the dominant tg rotamer. In a subsequent study, Chythra et al.^84^ used a revised Drude force field and reported a significantly improvement between simulations and experimental data. The root-mean-square error for ^3^J_H5,H6R_ was reduced from 3.8 to 1.6 Hz, and for ^3^J_H5,H6S_ from 1.7 to 1.3 Hz. These results underscore the significant improvements made with the updated Drude force field. In our AMOEBA study, we computed the dihedral angle ω for β-Glc.^23^ The computed distribution of the three rotamers also showed a deviation from the experimental data, with the simulations yielding 70(39)% gg, 28(22)% gt, and 2(39)% tg with­(out) mutual polarization. Note, however, that the time sampled in all of these studies ranges from 10 ns to 5 μs. This certainly contributes to differences in reported accuracy of the various force fields. Indeed, the rotation of hydroxymethyl groups is known to occur on relatively long time scale (nanoseconds to tens of ns) such that even the ideal force field may not adequately sample the appropriate gg, gt, and tg populations within ns MD simulations. As a result, the conformational populations may not fully converge, potentially affecting the accuracy of computed averages. In this case, a simple reweighing of the sampled conformers during postanalysis when computing average properties should easily address the sampling limitation. Finally, the discrepancy between different NMR data sets often surpasses discrepancies between theory and experiments,^85^ which complicates the direct optimization of force fields for this torsional parameter.

## Ring Puckering in Carbohydrates: Real or Force Field Artifact?

The six-membered ring in carbohydrates is known to undergo conformational changes, called ring puckering.^86^ The process, sometimes fleeting, occurs when the ring atoms pseudorotate in a coordinated manner. Chair (^4^C_1_ or ^1^C_4_) and boat (^1,4^B, ^2,5^B, ^3,0^B, B_1,4_, B_2,5_, B_3,0_) are the most visited conformations,^87^ but transitions between these minima involve sampling a complex conformational landscape comprising multiple boat or skew-boat conformer minima and envelope conformer transition states.^88–90^ Classical force fields all predict the ^4^C_1_ conformation to be the most stable for monosaccharides, including d-glucose. This is consistent with experimental studies for the d-series of aldohexopyranoses, in which the ^4^C_1_ chair is the predominant conformation.^91,92^

The Cremer-Pople internal coordinate system was derived to define the multitude of potential conformations.^93^ For a six-membered ring, three independent coordinates are used: the puckering amplitude or radius, *Q*, and two phase angles, θ that ranges from 0 to 180° and φ that ranges from 0 to 360°. *Q* is the sum of the perpendicular distance of each ring atom *j* to the ring average plane

2 $$Q=\overset{6}{\underset{j=1}{\sum }}z_{j}$$

and ϕ and θ are obtained by solving the following system of equations:

$$\{\begin{array}{l} Q\;\sin\left(\theta \right)\cos\left(\phi \right)=\sqrt{\frac{1}{3}}\overset{6}{\underset{j=1}{\sum }}z_{j}\;\cos\left(\frac{2\pi }{6}\left(2j-1\right)\right)\\ Q\;\sin\left(\theta \right)\sin\left(\phi \right)=\sqrt{\frac{1}{3}}\overset{6}{\underset{j=1}{\sum }}z_{j}\;\sin\left(\frac{2\pi }{6}\left(2j-1\right)\right)\\ Q\;\cos\left(\theta \right)=\sqrt{\frac{1}{6}}\overset{6}{\underset{j=1}{\sum }}{\left(-1\right)}^{j}z_{j} \end{array}$$ 3

*Q*, θ, and ϕ describe a sphere (Figure 3a) that can be projected from the north pole into the Stoddart diagram (Figure 3b), which simplifies the map between conformations.

**Figure 3:**
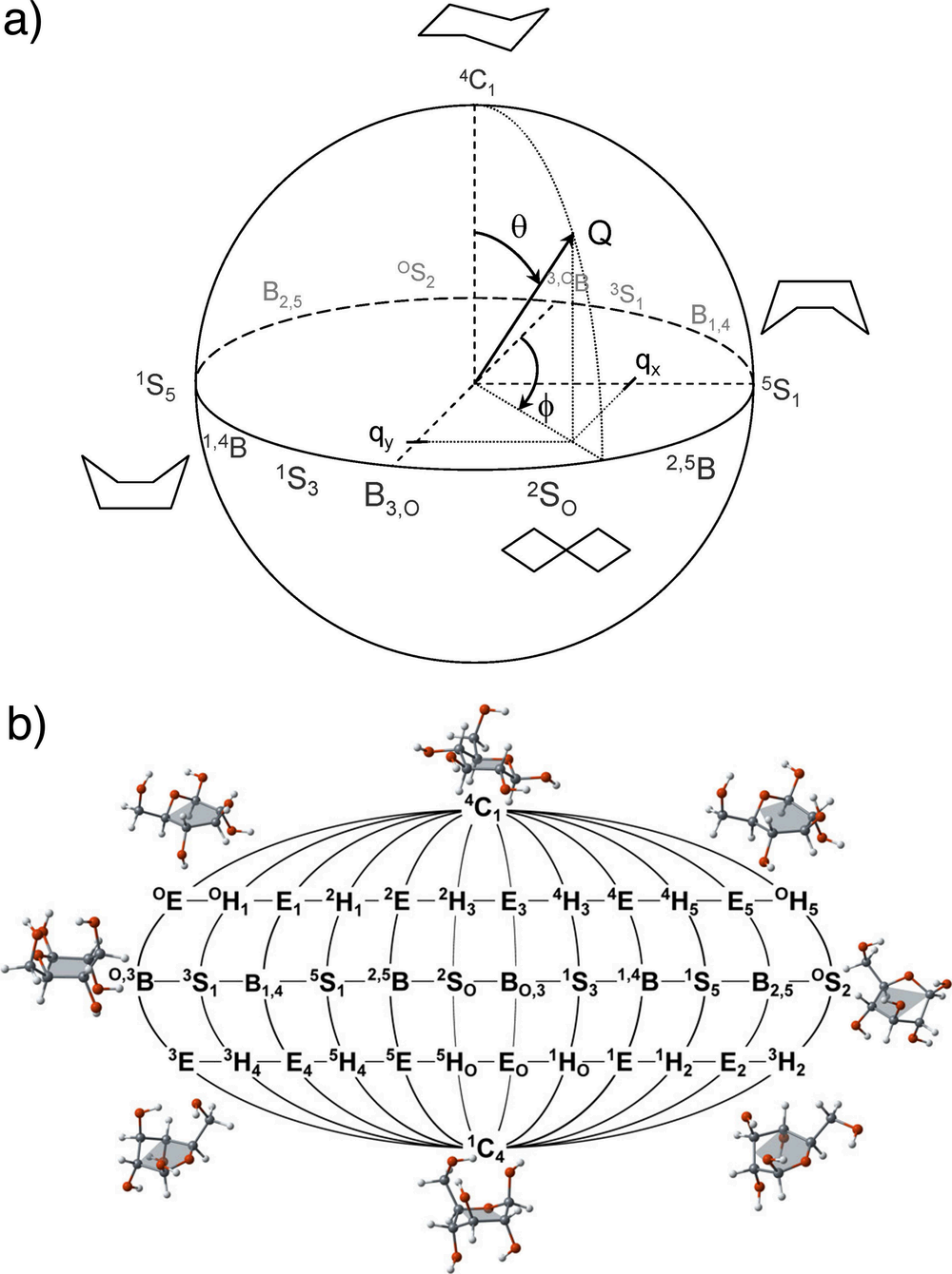
a) Cremer-Pople puckering coordinates representation and b) Stoddart diagram. Adapted with permission from ref 94.

Studies of ring puckering are difficult because most transitions occur over long time scales (microseconds), which is not trivial to simulate with atomistic models, even with enhanced sampling techniques.^95–97^ Further, it is difficult to validate because of the limited availability of experimental data, which relates only to the most stable conformations. Plazinski et al. compared four classical force fields—GROMOS 56A6_CARBO_, GROMOS 53A6_GLYC_, CHARMM, and GLYCAM06—for their predictions of ring inversion free energies of eight D-hexopyranoses monomers.^98^ They found that GROMOS 56a6_CARBO_ and CHARMM demonstrated good agreement with available experimental data and the semiempirical Angyal scheme. In contrast, GLYCAM06 showed the poorest agreement, while GROMOS 53a6GLYC captured qualitative trends with limited quantitative accuracy.^98^ The study also emphasized the importance of *ab initio* MD, often presumed more accurate, for benchmarking classical MD force fields due to the lack of comprehensive experimental reference data. For example, Biarnés et al. employed Car–Parrinello MD and metadynamics to map the free energy landscape of glucopyranose using the Cremer-Pople puckering coordinates as collective variables.^94^ The computed free energy landscape revealed eight local minima, in contrast to the 12 canonical conformations described in Stoddart’s diagram. Of the eight conformations, five aligned with canonical forms (B_3,O_, ^1^S_5_, B_2,5_, ^3,O^B, and B_1,4_), while the remaining three were intermediate states positioned between two standard conformations (e.g., B_3,O_/^2^S_O_, ^1,4^B/^1^S_3_, and ^2,5^B/^5^S_1_). Such deviations were attributed to two main factors: ring flexibility and the presence of intramolecular interactions among the OH ring substituents.

In a different study, Mayes et al. used *ab initio* MD to map the interconversion pathways between the different puckering geometries of β-xylose, β-mannose, α-glucose, β-glucose, and β-N-acetylglucosamine.^99^ The calculated potential energy surface of β-xylose showed that the most kinetically accessible conformation from ^4^C_1_ is ^2^S_O_, with a barrier height of 8.4 kcal/mol. Interestingly, such a state has been observed in NMR by Ronoola et al. along ^4^C_1_ and ^1^C_4_.^100^ This suggests that stable puckering geometries do not always conform to discrete IUPAC designations and that exocyclic groups significantly impact the kinetic landscapes of sugars. For example, the barrier height to leave the ^4^C_1_ energy well of oxane (without exocyclic group) is approximately 10 kcal/mol. However, the barrier heights for β-glucose to other puckering conformations range from 5 to 13 kcal/mol.^99^ Thus, the presence of exocyclic groups can either lower or increase the energy of transition states. Additionally, β-xylose and β-mannose exhibit different puckering behaviors due to their distinct exocyclic group arrangements, which affect their stability and interconversion pathways.^99^

This ever-changing landscape with multiple secondary minima observed in *ab initio* MD further complicates the validation of classical force fields. This is because possible intermediate states and MD artifacts from a bad force field can be difficult to distinguish. For example, the sampling of glucopyranose ring conformers with the GROMOS 45a4/SPC force field revealed four metastable conformations with increasing relative free energies: ^4^C_1_ (most stable), ^1^C_4_, ^3,O^B, and ^1^S_3_.^101^ The main conversion route was reported to go from ^4^C_1_ to ^3,O^B to ^1^C_4_, with ^1^S_3_ being the higher-energy state. Transitions from ^4^C_1_ occur over different time scales, with ^3,O^B forming within 15 ns, while ^1^C_4_ and ^1^S_3_ require 0.5–0.8 μs. However, when the authors compared their simulations in vacuum to quantum mechanical data,^102–105^ the ^1^C_4_ conformer was overstabilized by 3.6 kcal/mol, and the boat conformers were understabilized by 2.4 kcal/mol, suggesting that the time scales and interconversion pathways from GROMOS 45a4 may not be accurate.

Spiwok et al. compared the performance of the same GROMOS 45a4 force field to GLYCAM06 and OPLS for glucose.^106^ The GROMOS 45a4 force field led to the same four minima: ^4^C_1_, ^1^C_4_, ^1^S_3_, and ^3,O^B/^O^S_2_. However, they report stabilization of ^3,O^B/^O^S_2_ in water, making ^1^S_3_ more accessible from ^3,O^B/^O^S_2_ than from ^4^C_1_, resulting in only a qualitative agreement with the work of Hansen and Hunenberger.^101^ GLYCAM06, with AMBER 1–4 scaling, identified three minima: ^4^C_1_, ^2^S_O_, and ^1^C_4_. Removal of the 1–4 scaling introduced additional boat/skew-boat minima but did not significantly change the transition barriers. Meanwhile, the OPLS force field produced a free energy surface with six minima in water with relatively high energy barriers. Similarly, Wang and Berne found five intermediates, in addition to the two chair conformers, with the OPLS3/SPC force field: ^2^S_O_, B_O_, ^3,1^S_3_, ^2^H_1_, and ^5^S_,_ known to be relevant in biological processes.^107,108^

Although it is evident that these force fields do not accurately capture the key ring puckering intermediates for d-glucose, it is not trivial to know which force field is most accurate. Indeed, compared with other monosaccharides, d-glucose was reported to be significantly more flexible.

For example, some believe that the appearance of boat conformers for d-glucose may result from a minor deficiency of the force field in terms of the ring torsional potential. In this context, Hansen and Hunenberger reoptimized the GROMOS 53a6 force into a new version 56a_CARBO_ to study the free energies (Δ*G*) associated with the full chair–chair ring inversion from ^4^C_1_ to ^1^C_4_.^85^ Their Δ*G* compared relatively well with experimental and semiempirical data,^80,103,109,110^ though with reduced accuracy for β-altrose and β-idose. However, they concluded that the observed deviation for β-altrose and β-idose was due to the two-state model used in data fitting, and a direct comparison with the primary experimental data (i.e., J-coupling constants) showed good agreement. This underscores the importance of validating force field performance against primary experimental data whenever available. Others suggest that the transition to boat conformers occurs on a shorter time scale, which is harder to characterize experimentally. Plazinski and Drach determined the rate constants and dynamics of chair–chair and chair-boat transitions in d-glucose with GROMOS 56a_CARBO_, GLYCAM06, and GROMOS 53a6_GLYC_.^111^ Their findings indeed showed that the ^4^C_1_ to ^1^C_4_ transitions occur on time scales ranging from microseconds to milliseconds, while chair-boat transitions occur on a slightly shorter time scale. GROMOS 56a6_CARBO_ and GLYCAM predicted time scales in moderate agreement with experimental data. However, GROMOS 53a6_GLYC_ overestimated the free energy barriers and thus the time scales. This suggests that a simple reoptimization^112^ of these force fields is not sufficient and that they lack a fundamental term, possibly polarization.

Chythra and Sairam investigated the influence of polarizable force fields on the conformational dynamics of five hexose monosaccharides: glucose, mannose, galactose, altrose, and idose and their anomers.^78^ The additive CHARMM simulations were run for 5 μs and the polarizable Drude simulation was run for 2.5 μs. Analysis of their results revealed that the additive simulations predominantly sampled the ^4^C_1_ conformations for all sugars except for α-idose, while the polarizable force field sampled both chair as well as intermediate conformations. However, the authors observed several shortcomings with the Drude force field, such as oversampling of the ^1^C_4_ conformation for alpha-mannose (82%) and α-altrose (91%), which is inconsistent with experimental data.^113^ Note that these issues were not observed in another Drude investigation of the ring puckering and inversion properties of hexopyranose monosaccharides, probably because the MD were only run for 10 ns.^64^ To address this discrepancy, the authors in 2024 employed a revised version of the Drude polarizable carbohydrate force field.^84^ These new parameters improved the conformational sampling of the ^4^C_1_ conformations for mannose (60%) and α-glucose (99%) and ^1^C_4_ for α-idose (98%). However, the new data for α-altrose was still inaccurate when compared to experimental data. Overall, although the Drude_new_ parameter set represents a significant improvement, there are still areas that require further research and refinement to achieve more accurate modeling of certain hexopyranose monosaccharides. As force fields advance, there will be a greater consensus between simulations, which will help resolve the ambiguity raised by the lack of accessible experimental data.

## From Monosaccharides to Polysaccharides: Modeling Glycosidic Linkages

In the previous sections, we mostly discussed the modeling of monosaccharides in water. However, as force fields improve, the structure and dynamics of these carbohydrates can be modeled fairly accurately, and we can now turn to the modeling of polysaccharides. In this case, a precise model of glycosidic linkages is essential. Unlike peptide bonds in proteins, glycosidic linkages can occur from any position on the ring of one carbohydrate to any other position on the ring of another. The bonds are highly flexible and play a crucial role in hydration behavior, conformational changes, and the overall three-dimensional arrangement of polysaccharides. These structural characteristics, in turn, influence their functions, including their interactions with other biomolecules.^19,114–116^ The relative orientation of adjacent monosaccharide units is characterized by the glycosidic dihedral angles ϕ and ψ, which define the rotational freedom around the glycosidic bond. Note that the relative orientation of 1–6 linked sugars is also characterized by ω. Accurate capture of these dihedral angles is critical for reproducing experimental structural data and predicting polysaccharide properties.

For the most part, studies investigating the dynamic conformations of glycosidic linkages have relied on nonpolarizable force fields such as CHARMM,^62,117^ GROMOS,^85,118^ and GLYCAM.^52^ Salisburg et al. conducted implicit solvent MD simulations of eight disaccharides with β-d-(1 → 4), α-d-(1 → 3), and α-d-(1 → 6) equatorial linkages to validate the GLYCAM06 force field against X-ray crystallographic data.^119^ Their findings demonstrated a strong correlation between the simulated dihedral angles (ϕ, ψ, and ω) and experimental values.^120^ On average, the absolute differences between simulations and X-ray structures were 10.3°, 12.6°, and 14.0°, for ϕ, ψ, and ω, respectively. Meanwhile, deviations from available NMR data were 8.6° and 15.2° for ϕ and ψ, respectively. Overall, the results indicate the ability of GLYCAM06 to accurately model disaccharides conformations. This was mostly confirmed in another study although the authors indicated that GLYCAM06 tended to overlook higher-energy (ϕ, ψ) conformers.^121^ This discrepancy was due to the relaxation of these higher-energy states toward lower-energy conformations, potentially due to a reduced energy barrier between the two regions. However, these GLYCAM06 simulations were performed without explicit solvent, and the choice of water model in future simulations may change this outcome. In a more recent comparative analysis of the structural and thermodynamic properties associated with glycosidic linkages, notable differences were found among CHARMM, GLYCAM, and GROMOS 56a6 _CARBO_.^116^ Specifically, differences were observed in the relative energy landscapes of glycosidic torsional angles (anti-ϕ and ψ) across various linkage types and particularly pronounced deviations in the flexibility of β (1 → 3) conformers. Additional variations were observed in the hydrogen-bonding frequencies and in the conformational distributions of the hydroxymethyl group. Importantly, these differences were found to accumulate at larger structural scales, specifically in the persistence lengths of the polysaccharide chains. Note that the trends observed in this study did not lead to the identification of a “better” force field due to the lack of benchmark data.^98,116^ However, Balogh et al. found that CHARMM outperformed GLYCAM06 and GAFF1 in predicting the conformational flexibility of idraparinux (pentasaccharides) derivatives.^122^ This was attributed to a better torsion angle parameter for the sulfonatomethyl group in CHARMM. Overall, these findings emphasize the need to incorporate parameters that capture the conformational behavior of longer carbohydrate chains during force field development and refinement.

Further, most classical force fields are parametrized for simulations at ambient conditions, with limited emphasis on temperature dependence.^123,124^ A detailed investigation revealed that CHARMM C35, GLYCAM06, and GROMOS 56a6_CARBO_ force fields overestimate the temperature dependence of ω conformational populations relative to experimental NMR data, for both short and extended oligosaccharide chains.^124^ The extent of the discrepancy was found to be dependent on both the force field and the water model, with GLYCAM06 displaying the largest temperature dependence, followed by CHARMM. The TIP3P water model showed enhanced temperature dependence, with a reduction in SPC/E models. In contrast, experimental NMR data showed only minor temperature effects, highlighting the need for improvement in force fields to better capture temperature-dependent behavior in polysaccharides.^124^

Other MD simulations were performed to investigate the role of polarization in modeling glycosidic linkages with Drude/SWM4-NDP.^125^ A key advantage of the Drude force field is its ability to accurately represent cooperative intramolecular hydrogen bonding among three or more vicinal hydroxyl groups, a common feature in polysaccharides.^126^ To validate the force field against experimental crystal lattice parameters, simulations were performed using a (2 × 2 × 2) supercell with varying linkages and amount of sugar and water molecules. Compared to the CHARMM force field, Drude showed better agreement with the experimental crystallographic parameters of 41 compounds.^117,127–130^ Specifically, the average deviations from the experimental unit cell parameters were 1.35% (A), 0.85% (B), 1.69% (C), and 3.52% (volume) for the Drude force field, while CHARMM exhibited slightly higher deviations of 1.3%, 0.9%, 2.4%, and 4.7%, respectively. In addition, Drude yielded average deviations of 0.003 Å, 3.89°, and 13.3° for bond lengths, angles, and dihedral, respectively. Interestingly, while Drude outperformed CHARMM for most systems, CHARMM exhibited slightly better performance for polysaccharides containing pyranose, particularly for (1 → 6) linkages. This was attributed to specific optimizations applied during the parametrization, particularly with respect to ω and ^3^J-coupling constants.

Despite promising results for monosaccharides and disaccharides, the application of polarizable force fields in larger polysaccharides remains scarce. Our group is actively working on applying AMOEBA to polysaccharides, following our previous work on monosaccharides.^23^ AMOEBA predictions, combined with existing Drude predictions, will likely enable the identification of key factors to be included for the modeling of polysaccharides, thereby advancing the development of general force fields for carbohydrates.

## Conclusions

In this Perspective, we examined the application of various force fields for MD simulations of polysaccharides, highlighting their strengths and limitations. Although widely used, nonpolarizable force fields such as CHARMM, AMBER/GLYCAM, and GROMOS are only partially successful. More specifically, they tend to perform well for solute geometries but fail to adequately capture the complex interactions between carbohydrates and water. Our discussion underscores how polarizable force fields, such as Drude and AMOEBA, are better suited to the modeling of carbohydrates in solution. This result holds even when accounting for the larger computational cost associated with these advanced force fields, with AMOEBA being the most expensive. When necessary, enhanced sampling methods can be used to help converge specific properties with these force fields. Our discussion also emphasizes the importance of consistency in the development of force field parameter sets. This is important when introducing a new water model to an existing force field or when adjusting the van der Waals parameters. For example, the Drude force field required revisions of its nonbonded potential energy terms to better balance carbohydrate-water interactions with water–water interactions. In contrast, the recent success of AMOEBA in accurately modeling monosaccharide solution properties without revisions of the force field suggests that its potential energy functions fully capture the essence of bonded and nonbonded interactions in solution. Therefore, its application to polysaccharides represents a promising avenue for future research. Indeed, expanding the AMOEBA force field parameter sets to polysaccharides has the potential to significantly improve our understanding of their structural and dynamic behavior, particularly in aqueous or protein environments, where hydration effects play a fundamental role. A concise overview of the strengths and limitations of each force field in accurately modeling key carbohydrate properties is shown in Table 1.

**Table 1:** Force Field Predictive Capabilities with Respect to Hydration Properties (RDF Peak Position and Hydration Water Structure), Kinetics (Non-Physical Aggregation and Diffusion Coefficient), *ω* (Qualitative Population Ordering and Quantitative % of Populations), and Ring Inversion Properties (Sampling and Energy Barrier)^a^

	Hydration properties		Kinetics		ω (gg,gt,tg)		Ring Inversion	
Force field/water model	Peak position	Water structure	Agg	Diffusion	Order	% Pop.	Sampling	Δ*G*
AMBER/SPC/E	√	∼	–	–	–	–	–	–
GROMOS 45a4/SPC/E	–	∼	–	–	√	∼	∼	×
GROMOS 56A6_CARBO_/SPC/E	√	∼	×	×	√	∼	√	√
GROMOS 53A6_GLYC_/SPC/E	–	–	–	–	√	∼	√	√
GLYCAM06/TIP3P	√	∼	×	×	√	√	√	×
CHARMM/TIP3P	√	∼	×	×	√	∼	√	√
Drude/SWM4-NDP	√	√	×	∼	√	∼	√	×
AMOEBA/AMOEBA	√	√	√	√	∼	×	–	–

a Note that the predictive capabilities of the force fields depend on the water model. We list the most commonly used water model for each force field (i.e., those for which we had the most data). Δ*G* is the free energy associated with the full chair–chair ring inversion from ^4^C_1_ to ^1^C_4_. Consistent with experimental or high-level data (√); inconsistent (×); partially consistent or ambiguous (∼); Data not available or not evaluated (−).
